# Pediatric-Type Diffuse Low-Grade Gliomas: A Subgroup Defined by Peculiar Molecular Features and Distinct Prognostic Outcomes

**DOI:** 10.7759/cureus.88575

**Published:** 2025-07-23

**Authors:** Abeer Javed

**Affiliations:** 1 Pathology, Akhtar Saeed Medical & Dental College, Rawalpindi, PAK

**Keywords:** brain neoplasms, classification, clinical trial, molecular diagnostics, molecular targeted therapy

## Abstract

Despite their biological and molecular heterogeneity, pediatric-type diffuse low-grade gliomas exhibit significantly different prognostic outcomes compared to their adult-type counterparts. Accurate diagnosis is essential to avoid aggressive overtreatment and to enable exploration of relevant molecular targets for personalized therapy. This review provides a comprehensive overview of the epidemiology, clinical presentation, radiologic findings, histopathologic features, and key molecular events characterizing the newly defined WHO subgroup "pediatric-type diffuse low-grade gliomas." The review also outlines the conventional treatment modalities, including surgery, chemotherapy, and radiotherapy, while discussing their limitations and adverse effects. In addition, emerging therapeutic strategies based on molecular targets are briefly highlighted, offering a glimpse into current clinical trials and FDA-approved targeted therapies. Data were retrieved from credible scientific sources including PubMed, Google Scholar, and the 2021 WHO Classification of Central Nervous System Tumors. The newly established molecular subgroup comprises four distinct entities: 1) Diffuse astrocytoma, MYB or MYBL1-altered; 2) Angiocentric glioma; 3) Polymorphous low-grade neuroepithelial tumor of the young; and 4) Diffuse low-grade glioma, MAPK pathway-altered. Unlike circumscribed astrocytic gliomas, these tumors exhibit partial infiltrative behavior. However, they tend to have a more favorable prognosis than adult-type diffuse gliomas, IDH-mutant. Circumscribed gliomas are typically managed with gross total resection and show a lower recurrence rate in comparison to this newly recognized subgroup. While surgical resection remains curative for small, superficial tumors, deeper or more infiltrative variants may recur following subtotal resection. A thorough understanding of the clinicopathological and molecular features of these gliomas is imperative for accurate classification and appropriate therapeutic intervention.

## Introduction and background

Central nervous system (CNS) tumors, including brain tumors, are the most common type of solid tumor and the leading cause of cancer-related deaths among individuals aged 0-19 years [1]. CNS tumors result in 0.7 deaths per 100,000 children diagnosed, establishing them as the leading cause of cancer-related mortality in the pediatric population [2,3]. Within this category of central nervous system tumors, gliomas alone account for 50% of the cases [4,5]. In adults, high-grade gliomas are more prevalent; however, low-grade gliomas predominate in the pediatric age group [4,5].

Pediatric low-grade gliomas account for approximately 30% of childhood CNS tumors. These are classified as World Health Organization (WHO) grade I or II tumors and encompass a diverse range of histological subtypes that can arise throughout the neuroaxis [6,7]. Pediatric low-grade gliomas are classified into three distinct subgroups: circumscribed astrocytic gliomas, glioneuronal and neuronal tumors, and the recently defined pediatric-type diffuse low-grade gliomas [4].

Pediatric-type diffuse low-grade gliomas, like their adult counterparts, have an infiltrative border. The formation of secondary structures of Scherer is a consistent feature that renders the acquisition of clear surgical margins difficult, if not impossible, in certain deep-seated tumors [8]. A unique subset of pediatric diffuse gliomas carrying the IDH gene mutation was highlighted by Ferris et al. In their study, whole-exome sequencing identified three cases of IDH-mutant diffuse gliomas in pediatric patients. However, these were thought to be genetically unique from adult-type tumors, since they were ATRX non-mutated and lacked TERT promoter mutations [9]. This review discusses the clinicopathologic and molecular features of pediatric-type diffuse low-grade gliomas, current treatment limitations, and emerging therapies to enhance understanding, support accurate diagnosis, and guide precise management.

## Review

Pediatric-type diffuse low-grade gliomas

Diffuse astrocytoma, MYB- or MYBL1-altered, CNS WHO grade 1, is a newly recognized tumor entity in the 2021 World Health Organization classification of central nervous system tumors. Diagnosis requires an integrated approach, considering both histologic and molecular features, in keeping with the 2014 ISN-Haarlem guidelines and C-IMPACT-NOW updates [10,11].

Diagnostic Criteria

Diffuse astrocytoma, MYB- or MYBL1-altered, is a diffusely infiltrative astrocytic neoplasm that lacks histomorphological features indicative of an anaplastic nature [7]. For a definitive diagnosis, the tumor must be IDH-wild type and H3-wild type, and should exhibit a structural variant involving either the MYB or MYBL1 gene. In cases where such genetic alterations are not detectable, a distinct DNA methylation profile characteristic of diffuse astrocytoma, MYB- or MYBL1-altered, may be sufficient to establish the diagnosis. This rare tumor entity represents approximately 2% of the pediatric low-grade glioma spectrum, and accounts for only 0.3% of all tumors surgically removed in the context of epilepsy management [12-14].

These histologically indolent-appearing tumors often present in pediatric patients with persistent, treatment-resistant epileptic seizures. As a result, along with glioneuronal tumors, they are classified within the broader clinical category of long-term epilepsy-associated tumors (LEATs). In the largest case series published by Chiang et al. comprising 46 gliomas from St. Jude’s Hospital, the median age of presentation was 5 years, and no gender predilection was observed. The majority of the tumors were localized in the cerebral cortex, followed by the cerebral white matter/deep gray nuclei or brainstem. Epilepsy was the most frequent presentation of tumors located in cortical lobes; raised intracranial pressure in neoplasms involving the white matter/deep gray nuclei, whereas multiple cranial nerve deficits were observed in brainstem tumors [14].

Given the tumor’s low-grade, infiltrative histologic features, the radiologic findings are consistent with those of an indolent glioma. Preoperative magnetic resonance (MR) images reveal a T1 iso-intense to hypo-intense tumor with a mixed signal or hyperintensity on T2-FLAIR. These tumors are mostly well-delineated, although some may exhibit a diffuse growth pattern. In rare cases, large cysts may also be present [15,16].

**Figure 1:**
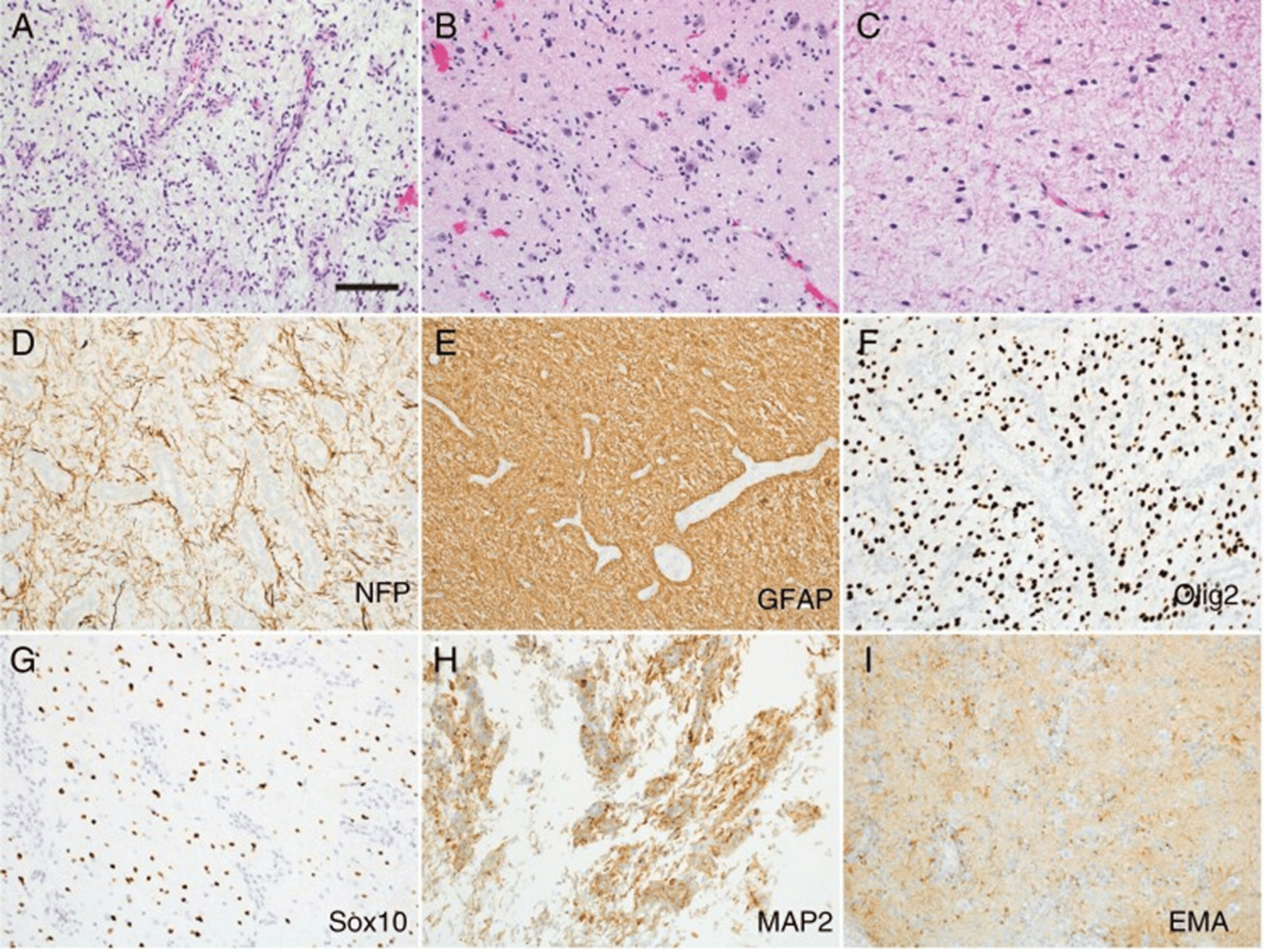
Histopathology of MYB/MYBL1-altered pediatric-type diffuse low-grade glioma. (A) Tumors with an angiocentric glioma pattern exhibit prominent angiocentric growth and areas resembling diffuse astrocytoma. Entrapped neurons are visible. (B) Tumors with a diffuse astrocytoma pattern often display varying degrees of angiocentric growth around small blood vessels. (C) Isomorphic diffuse glioma reveals subtle increases in cellularity. (D) The tumor shows widespread infiltration of CNS parenchyma, with numerous entrapped neurofilament-positive axons. (E) Tumor cells demonstrate GFAP positivity. (F,G) Tumor cells with angiocentric growth are negative for Olig2 and Sox10. (H) Angiocentric tumor cells show MAP2 immunoreactivity. (I) A characteristic finding is the perinuclear dot-like EMA immunoreactivity. Scale bar: 100 μm. Image Source: Moreira et al. [17], shared under the Creative Commons CC-BY-NC license. CNS: central nervous system; GFAP: Glial fibrillary acidic protein; EMA: Epithelial membrane antigen; MAP2: Microtubule-associated protein 2; Olig2: Oligodendrocyte transcription factor 2

The tumor exhibits characteristic histology of a low-grade astroglial neoplasm with a diffusely infiltrative border. Aggressive histologic features such as overt nuclear pleomorphism, brisk mitotic activity, microvascular proliferation, and necrosis are sometimes typically absent. Both subpial condensation and a diffusely infiltrative growth pattern were consistently observed histologic features of the neoplasm. Occasionally, tumor cells exhibited a subtle angiocentric pattern of growth. The characteristic molecular profile can be investigated using multiple ancillary studies, including immunohistochemistry, RNA sequencing, interphase FISH, and qualitative PCR-based sequencing [12]. These tumors are specifically characterized by MYB or MYBL1 alterations and a lack of mutations in IDH1, IDH2, TP53, ATRX, and H3 genes. Methylation studies using t-distributed stochastic neighbor embedding (t-SNE) analysis identified a single cluster, regardless of tumor site, neuroradiologic findings, or histopathologic features, indicating that this neoplasm is molecularly distinct despite overlapping clinicopathologic characteristics (Figure 1) [14].

Angiocentric glioma, CNS WHO grade 1

Angiocentric glioma is a relatively rare, indolent astrocytic tumor first described in 2005 in two different studies by Wang et al. and Lellouch-Tubiana [18,19]. It was then formally incorporated as a distinct tumor entity in the WHO classification of tumors of the central nervous system in 2007 [16].

WHO Diagnostic Criteria

Essential Features: Angiocentric glioma is a low-grade glial neoplasm with a focal angiocentric pattern of growth and a predominant solid or diffuse architecture. These tumors comprising monotonous spindle cells exhibiting immunohistochemical and ultrastructural features consistent with an astrocytoma or ependymoma.

Desirable Features: Angiocentric glioma is a diffusely infiltrative, low-grade glioma characterized by several desirable histological and molecular features. Notably, it shows no evidence of anaplasia and often harbors MYB alterations, with a DNA methylation profile closely resembling that of diffuse glioma, MYB- or MYBL1-altered. This tumor affects individuals across a wide age range, from infancy to over 80 years, with a median age of 15 years and a slight male predominance [20]. Childhood-onset, drug-refractory seizures are the most common presenting feature; however, patients may exhibit a wide array of symptoms including headaches, cranial nerve deficits, generalized weakness, nausea, vomiting, gait disturbances, nystagmus, and spinal impairment [21].

On MRI, angiocentric gliomas typically appear as unifocal, well-delineated, superficial supratentorial cortical tumors that are T1 hypointense and T2-FLAIR hyperintense. Most tumors are non-enhancing and display a characteristic “stalk sign”-a stalk-like extension into the lateral ventricles. Although uncommon, brainstem and thalamic involvement has been documented, often associated with a distinct molecular profile. Other radiologic findings may include restricted brain atrophy, calcification, prominent cystoid changes, and a rich vascular supply [22].

Histologically, these tumors demonstrate a unique angiocentric growth pattern with bland, uniform bipolar spindle cells arranged concentrically around blood vessels. Cellular pleomorphism, anisonucleosis, microvascular proliferation, necrosis, and mitotic activity are generally absent. Some cases may exhibit nuclear palisading, myxoid changes, or calcifications. The tumor typically shows subpial condensation, with nuclei aligned almost perpendicularly to the pia mater. Interspersed neurons are consistently observed; whether they are neoplastic or represent entrapped cortical neurons remains unclear [18, 23]. Focal cortical dysplasia has been noted in the adjacent cortex in several cases [20].

Immunohistochemically, tumor cells consistently express GFAP, S100, and vimentin, with a distinctive perinuclear dot-like EMA positivity. They are negative for IDH1, IDH2, p53, Olig2, and NeuN, and the Ki-67 proliferation index remains low, typically under 5%, consistent with WHO Grade I status [19].

Molecularly, nearly all tumors exhibit gene rearrangements or copy number alterations involving the MYB locus on chromosome 6q23 [24]. 3. The most consistent genetic alteration is the MYB: QKI fusion, involving the proto-oncogene MYB and QKI, a gene encoding an RNA-binding protein crucial for oligodendrocyte and Schwann cell development. Although rare, fusion partners other than QKI have also been identified [25]. Importantly, mutations commonly seen in higher-grade gliomas, such as IDH1, IDH2, ATRX, TP53, and H3, are absent in angiocentric gliomas. The DNA methylation profile of this tumor closely aligns with that of diffuse astrocytoma, MYB- or MYBL1-altered [26].

**Figure 2:**
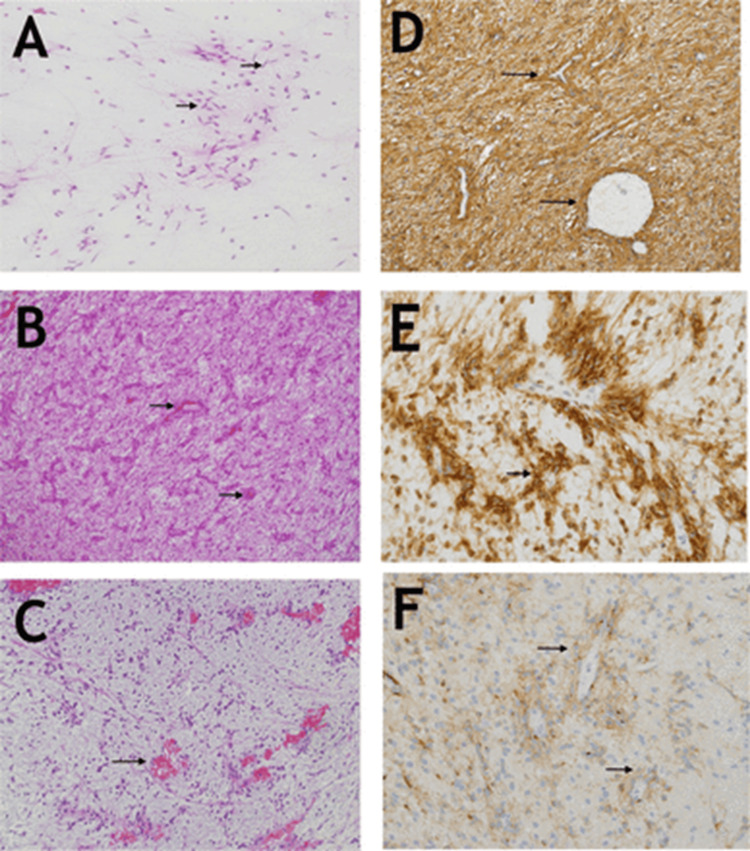
Angiocentric glioma, CNS WHO Grade 1 (A) Smear preparation reveals bipolar cells (arrows) with long processes, resembling pilocytic astrocytoma (Hematoxylin and Eosin, 200x). (B) At low magnification, the tumor shows mostly parallel neoplastic bipolar cells arranged in a distinctive angiocentric pattern, (arrows) (H&E, 100x). (C) The myxoid component is markedly hypocellular but retains the angiocentric configuration (arrows) (H&E, 100x). (D) Tumor cells show diffuse GFAP expression, particularly highlighting the angiocentric pattern (GFAP, polyclonal, Ventana, 200x). (E) Strong D2-40 expression is seen in tumor cells (arrows) (clone D2-4, DakoCytomation, 400x). (F) EMA shows moderate cytoplasmic staining, with perinuclear accentuation noted in some cells (arrows) (EMA, clone E29, Dako, 400x). Image Source: Almubarak et al. [27] shared under the Creative Commons CC BY-NC license GFAP: Glial fibrillary acidic protein; EMA: Epithelial membrane antigen

Polymorphous low-grade neuroepithelial tumor of the young (PLNTY)

Polymorphous low-grade neuroepithelial tumor of the young (PLNTY) occurs predominantly in the pediatric age group as initially described in the pilot case series. However, with subsequent new case reports, tumors in the adult population also became evident [21].

 WHO Diagnostic Criteria

The WHO diagnostic criteria for diffuse glioma, MAPK-pathway altered, include several distinct features: 1) the tumor must be a low-grade glial neoplasm exhibiting at least focal infiltrative morphology; 2) there should be oligodendroglioma-like histomorphological features present in at least part of the tumor; 3) the tumor must demonstrate low mitotic activity, consistent with its low-grade nature [28]. Another essential criterion is the diffuse aberrant expression of CD34 throughout the tumor. Additionally, the tumor must be IDH-wild type, indicating the absence of IDH mutations [29]. Finally, there must be unequivocal evidence of a mutation in BRAF or FGFR2 or FGFR3, or in any of the other genes involved in the MAPK signaling pathway. The majority of tumors exhibit a variable degrees of calcification ranging from small calcospherules to large confluent calcifications, which, in addition to 1p/19q wild-type status, are a desirable diagnostic criterion for this entity [30].

PLNTY occurs predominantly in the pediatric age group as initially described in the pilot case series; however, with subsequent new case reports, tumors in the adult population also became evident [31]. Tumors tend to occur over a wide age range (4-57 years) without any gender predilection. As with other low-grade glioneuronal and glial neoplasms occurring in the temporal lobe, epilepsy is the most common presenting feature, followed by headache and visual disturbances. The temporal lobe is the most common location, followed by the occipital and frontal lobes [32].

Typical radiologic features include a temporal lobe cortical or subcortical mass lesion with well-delineated borders (on T1- and T2-weighted MR images), confluent calcifications (occasionally prominent central calcification with small peripheral scattered calcifications), and a mixed solid-cystic appearance. Calcifications are most evident on a computed tomography (CT) scan. The heterogeneous signal intensity on T1- and T2-weighted MR images is due to hypointensity in the region of confluent calcifications and hyperintensity in the peripheral regions. Occasionally, the tumor presents with edema or mass effect [33,34].

As the name suggests, these tumors exhibit polymorphous morphology, with both astroglial and oligodendroglioma-like features, the latter being more commonly observed in most cases. Tumors with spindle cells, pleomorphic cells, and ependymoma-type rosettes have also been reported. However, aggressive histomorphologic features such as markedly increased cellularity, microvascular proliferation, brisk mitotic activity, atypical mitotic figures, or areas of geographic tumor necrosis are, by definition, absent [35]. A hallmark of these tumors is calcification, ranging from tiny calcospherules to chunky, confluent, radiologically and histologically prominent calcifications. Characteristic features of low-grade circumscribed astrocytic neoplasms, such as Rosenthal fibers, myxoid microcysts, and eosinophilic granular bodies, are typically absent, as is the presence of dysmorphic neurons and ependymal rosettes, which are seen in glioneuronal tumors and ependymomas, respectively [36].

PLNTYs are negative for IDH1 and IDH2 immunomarkers. Diffuse immunoreactivity for Olig2 and GFAP, along with retained ATRX, as well as strong expression of CD34, is a characteristic finding. CD34 is also positive in several other low-grade epilepsy-associated neuroepithelial tumors (LEATs) such as gangliogliomas, DNETs, pleomorphic xanthoastrocytomas, and areas of focal cortical dysplasia. However, CD34 is particularly useful in differentiating PLNTY from oligodendrogliomas, which are CD34-negative [37].

**Figure 3:**
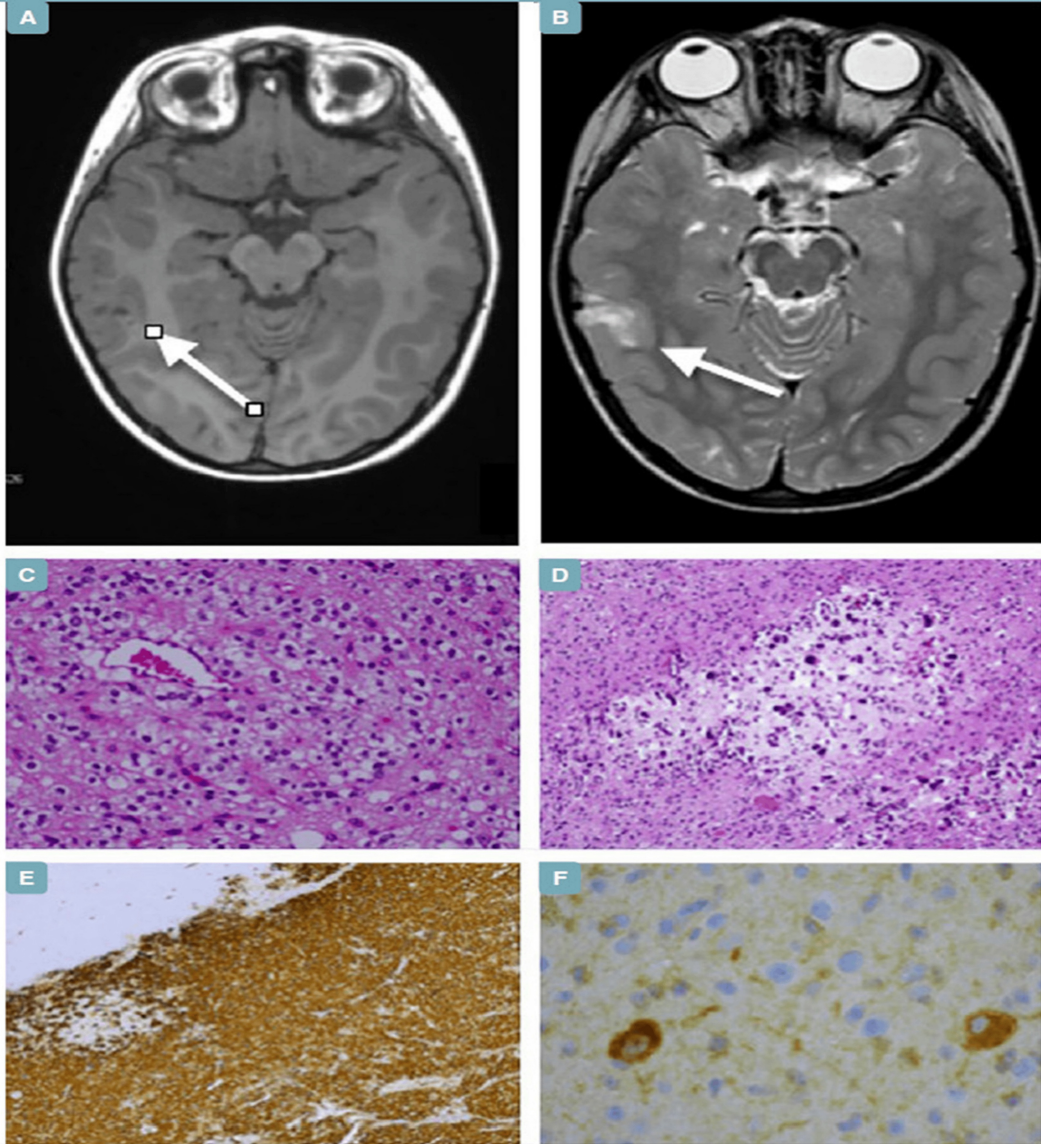
Polymorphous low-grade neuroepithelial tumor of the young, CNS WHO Grade 1 (A) T1-weighted imaging (T1WI) shows hypointensity. (B) T2-weighted imaging (T2WI) demonstrates hyperintensity with a “salt and pepper” appearance. (C) Histologically, the tumor consists of oligodendroglioma-like cells with interspersed thin-walled capillary channels (H&E, 20x). (D) Abundant microcalcifications are present within the lesion (H&E, 10x). (E) Tumor cells show intense and widespread CD34 immunopositivity (CD34, 4x). (F) BRAF V600E mutation is expressed in neoplastic cells (BRAF V600E, 40x). Image Source: Fabbri et al. [40] shared under the under a CC-BY-NC-ND 4.0 International License.

MAPK-pathway alterations are a consistent feature of polymorphous low-grade neuroepithelial tumor of the young. IDH1 and IDH2 mutations, as well as 1p/19q co-deletions, are absent in these tumors [38]. Mutations involving BRAF p.V600E are the most common, followed by alterations in FGFR2 or FGFR3 (both being mutually exclusive). FGFR2:SHTN1, FGFR2:INA, and FGFR3:TACC3 fusions have been identified using RNA sequencing. FGFR:CTNNA3 gene fusion, which is exclusive to this tumor, has also been recently reported, as has partial duplication of NTRK2 in a single case. PLNTYs display a DNA methylation profile clustering close to gangliogliomas [39] (Figure 3).

Diffuse glioma, MAPK-pathway altered

Diffuse glioma, MAPK-pathway altered, is a newly recognized tumor entity included in the WHO 2021 classification and defined by strict diagnostic criteria [1].

WHO diagnostic criteria

This entity refers to an infiltrative low-grade glial neoplasm exhibiting morphologic features of oligodendroglioma, astrocytoma, or mixed histology. It must exhibit either a fibroblast growth factor receptor 1 tyrosine kinase domain internal tandem duplication, a fibroblast growth factor receptor 1 mutation, or a mutation in BRAF p.V600E. In contrast, IDH and histone gene mutations, as well as CDKN2A deletions, must be absent. The majority of these tumors occur in the pediatric population, with no definite gender predilection. Although they can arise throughout the craniospinal neuroaxis, the cerebral hemispheres are the typical site, particularly the temporal lobe, similar to other low-grade epileptogenic neoplasms [38]. Radiologically, the tumor appears as a T2-FLAIR and T2 hyperintense, non-enhancing mass within the cerebral cortex, most frequently in the temporal lobe [41].

**Figure 4:**
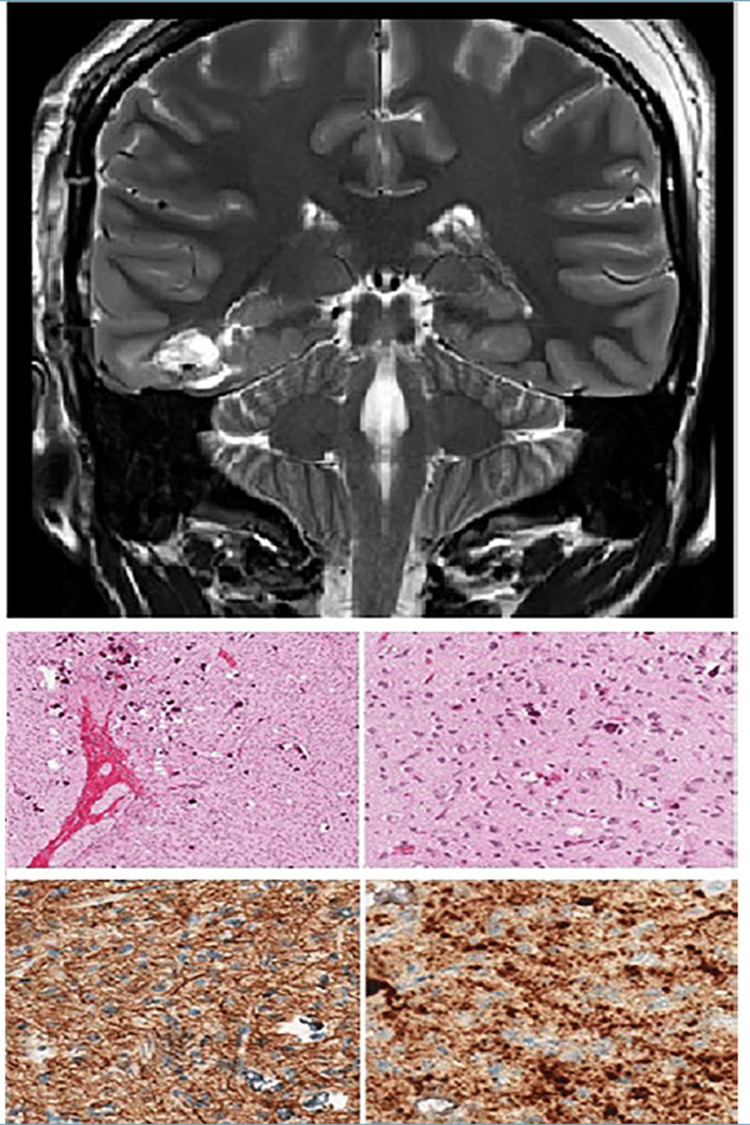
Diffuse glioma, MAPK-pathway altered (A) MRI shows a subcortical lesion with inhomogeneous signal and poorly defined margins. (B) Histology reveals neoplastic astrocytes entrapping cortical neurons and containing microcalcifications (H&E, 10x). (C) GFAP immunostaining demonstrates strong positivity (GFAP, ×20). (D) BRAFV600E immunoreactivity is present in tumor cells (BRAF, 20x). Image Source: Fabbri et al. [40] shared under the under a CC-BY-NC-ND 4.0 International License. MRI: Magnetic resonance imaging; GFAP: Glial fibrillary acidic protein

Histopathologic examination of these tumors reveals features characteristic of a low-grade infiltrative glial neoplasm. Tumors may exhibit morphology resembling pure oligodendroglioma or pure astrocytoma, while some show mixed histologic patterns. IDH-mutant diffuse gliomas in adults exhibit widely infiltrative tumor margins, which render the achievement of clear surgical margins cumbersome if not impossible. In contrast, diffuse low-grade glioma (MAPK pathway altered) shares morphologic features with other pediatric diffuse (non-IDH-mutant) low-grade gliomas and, therefore, displays minimal infiltration of the surrounding cortex, much less than adult-type IDH-mutated gliomas but more than pediatric circumscribed gliomas (such as pilocytic astrocytoma and pleomorphic xanthoastrocytoma) [42]. Features suggestive of aggressive biologic behavior, such as increased tumor cellularity, cellular pleomorphism, microvascular proliferation, and coagulative tumor cell necrosis, are not features of this tumor. Subpial condensation has been reported in certain cases. Interestingly, tumors characterized by FGFR1 alteration exhibit oligodendroglioma-like morphology and overlapping features with dysembryoplastic neuroepithelial tumors. These are frequently multinodular and exhibit clear cells with rounded nuclei and inconspicuous nucleoli. Relevant differential diagnostic considerations for this histologic appearance and molecular subtype include polymorphous low-grade neuroepithelial tumor of the young, which consistently shows diffuse non-vascular expression of CD34; adult-type diffuse astrocytoma and oligodendroglioma, which harbor characteristic 1p/19q co-deletion [28]. Conversely, BRAF p.V600E-mutated tumors reveal a fibrillary background with elongated to spindled cells showing mild to moderate cytologic atypia. Subpial condensation of the tumor cells may be seen. Pilocytic astrocytoma, pleomorphic xanthoastrocytoma, ganglioglioma, IDH-mutant gliomas, and diffuse midline glioma H3K27M-altered are included in the differential diagnoses. Low-grade circumscribed gliomas are easily distinguished based on the presence of Rosenthal fibers and eosinophilic granular bodies [43]. Gangliogliomas harbor dysplastic neuronal elements, separating them into a distinct category of their own. Immunohistochemistry and/or molecular testing conclusively excludes the presence of IDH and histone gene mutations, ruling out the last two differential diagnoses [1,44] (Table 1 and Figure 4).

**Table 1:** Immunophenotypic features and differential diagnoses of pediatric-type diffuse low-grade gliomas MYB: V-myb avian myeloblastosis viral oncogene homolog; MYBL1: V-myb avian myeloblastosis viral oncogene homolog-like 1; IDH1: Isocitrate dehydrogenase 1; GFAP: Glial fibrillary acidic protein; ATRX: Alpha-Thalassemia mental retardation syndrome X-linked; BRAF: B-Raf proto-oncogene, serine/threonine kinase; Olig-2: Oligodendrocyte lineage transcription factor 2; EMA: Epithelial membrane antigen; CD34: Cluster of Differentiation 34, MN-1: Meningioma 1 proto-oncogene; H3F3A G34R/V: H3.3 histone A, mutation where glycine at position 34 is replaced by arginine [R] or valine [V]; H3K27M: surrogate immunomarker for mutation in H3F3A or HIST1H3B/C gene where lysine 27 is replaced by methionine; DNET: Dysembryoplastic neuroepithelial tumor

Tumor Type	Marker	Result/Status	Differentials
Diffuse Astrocytoma, MYB/MYBL1-Altered	IDH1 p.R132H	Negative	Astrocytoma, IDH-mutant
	GFAP	Positive	Astrocytoma, IDH-wild type
	ATRX	Retained expression	Angiocentric glioma
	EMA	Dot-like intracytoplasmic [14]	Ganglioglioma
	CD34	Negative	CD34-negative gliomas
	Ki-67 Proliferation Index	< 1%	Low proliferative index gliomas
Angiocentric Glioma	IDH1 p.R132H	Negative	Astroblastoma, MN-1, altered
	GFAP	Positive	Astrocytoma, IDH-mutant
	EMA	Perinuclear dot-like	Astrocytoma, IDH-wild type
	Olig-2	Negative	Ependymoma
	Ki-67 Proliferation Index	< 1–5% [16,18,20]	Low-grade glioma
Polymorphous Low-Grade Neuroepithelial Tumor of the Young (PLNTY)	IDH1 p.R132H	Negative	Astroblastoma, MN-1, altered
	GFAP	Positive	Astrocytoma, IDH-mutant
	Olig-2	Positive	Astrocytoma, IDH-wild type
	CD34	Positive	Ependymoma
	ATRX	Retained expression	ATRX-retained gliomas
	BRAFp.V600E	Positive (in up to 54% of cases)[19, 45-48]	BRAF-mutant low-grade gliomas
Diffuse Low-Grade Glioma, MAPK Pathway-Altered	IDH1 p.R132H	Negative	Pilocytic astrocytoma
	GFAP	Positive	DNET
	Olig-2	Positive	Ganglioglioma
	BRAFp.V600E	Positive	BRAF-driven tumors
	H3F3A G34R/V	Negative	Excludes G34R/V mutant gliomas
	H3K27M	Negative	Excludes midline glioma, H3K27-altered

Conventional therapeutic strategies for pediatric-type diffuse low-grade gliomas

Tumor resection proves to be an effective treatment for almost all circumscribed astrocytic gliomas, conferring an excellent prognosis with a minimal recurrence rate [49,50]. However, with pediatric-type diffuse low-grade gliomas, achieving clear surgical margins is not always possible, and hence adjuvant chemoradiation is the treatment of choice (especially for tumors occurring in deep-seated cortex or the midline structures). Surgical outcomes depend upon various factors, including the degree of infiltration of the tumor, its location in the cortex, and histologic grade. Grade II/ Diffuse pediatric gliomas that are located in deeper cortex or involve midline structures pose a significant surgical challenge [51,52].

Complete resection may not be achievable due to the infiltrative nature of these tumors and their frequent involvement of eloquent or inaccessible brain regions. Approved initial chemotherapy regimens include carboplatin and vincristine (CV); four-agent therapy including thioguanine, procarbazine, lomustine, and vincristine (TPCV); vinblastine alone therapy; and carboplatin monotherapy [53-55]. Secondary malignancy, liver dysfunction, bone marrow failure, anaphylactic shock, and other allergies are noteworthy side effects of chemotherapy [53, 56].

Radiotherapy

Adjuvant radiotherapy, along with the cytotoxic conventional chemotherapy, had long been a standard of care for patients with recurrent or partially resected gliomas [57-60]. Unfortunately, conventional radiotherapy has caused several neurocognitive sequelae [61-64]. Recently, proton beam radiotherapy and stereotactically guided conformal radiotherapy have shown promising results concerning the safety profile [59,60] (Table 2).

**Table 2:** Molecular profile and therapeutic modalities for the treatment of pediatric-type diffuse low-grade gliomas MYB: V-myb avian myeloblastosis viral oncogene homolog; MYBL1: V-myb avian myeloblastosis viral oncogene homolog-like 1; QKI: QKI, KH domain containing RNA binding); PCDHGA1: Protocadherin gamma subfamily A, 1; MMP16: Matrix metallopeptidase 16; MME: Membrane metalloendopeptidase; MAML2: Mastermind-like transcriptional coactivator 2; LOC105378099: an RNA gene, affiliated with the ncRNA class [56]; BRAFp.V600E: BRAF proto-oncogene, serine/threonine kinase, V600E refers to a specific mutation; FGFR: Fibroblast growth factor receptor; KIAA1549 (a protein-coding gene in *Homo sapiens*; SHTN1: Shootin 1; INA: Internexin neuronal intermediate filament protein alpha; TACC3: Transforming acidic coiled-coil containing protein 3; CTNNA3: Catenin alpha 3; FGFR(TKD)ITD: Fibroblast growth factor receptor tyrosine-kinase domain internal tandem duplications; NTRK: Neurotrophic tyrosine receptor kinase; MET: MET proto-oncogene, receptor tyrosine kinase; MAP2K1: Mitogen-activated protein kinase kinase 1; MEK: Mitogen-activated protein kinase kinase; mTOR: Mammalian target of rapamycin; AZD4547: An inhibitor of the FGFR family; LOC154902: Rare fusion partner of MYB gene in diffuse astrocytoma, MYB/MYBL1-altered [73].

Tumor type	Molecular profile	Conventional treatment	Emerging therapies
Diffuse astrocytoma, MYB/MYBL1- Altered diffuse astrocytoma, MYB or MYBL1 altered	MYB/MYBL1 alterations (amplifications & gene fusions), fusion partners (QKI, PCDHGA1, MMP16, MME, MAML2, LOC105378099, LOC154902)	Gross total resection / maximum safe resection, focal radiotherapy, adjuvant conventional chemotherapy.	MEK Inhibitors (trametinib, selumetinib) (NCT03363217), mTOR inhibitors, MYB-targeted therapies [65-67]
Angiocentric glioma	MYB::QKI gene fusion [68-71]	Gross total resection / maximum safe resection.	MEK inhibitors (trametinib, selumetinib) (NCT03363217)
Polymorphous low-grade neuroepithelial tumor of the young	BRAFp.V600E & FGFR mutations (FGFR2, FGFR3), fusions (KIAA1549::BRAF, FGFR2: SHTN1, FGFR2: INA and FGFR3: TACC3, FGFR: CTNNA3)	Gross total resection, subtotal resection [19]	BRAF inhibitors (vemurafenib, dabrafenib) FGFR inhibitors (erdafitinib, AZD4547) [72-74]
Diffuse low-grade glioma, MAPK pathway-altered	BRAFp.V600E mutations, FGFR1 (TKD) duplications, FGFR1 hotspot mutations, NTRK1/2/3, MET, FGFR2 & MAP2K1 mutations.	Gross total resection, selective total resection.	BRAF inhibitors (trametinib, dabrafenib), MEK inhibitors (selumetinib), FGFR inhibitors (erdafitinib)

Limitations

Stereotactically-guided radiotherapy and proton beam radiotherapy are not yet widely accessible due to financial constraints and the requirement for specialized expertise. With the inevitable advancement in molecular pathology, several molecular targets have been identified, paving the way for the exploration of small-molecule inhibitors that cause fewer cytotoxic and radiotherapy-related adverse effects. This progress has led to numerous ongoing clinical trials and even the approval of several FDA-sanctioned targeted therapies for the treatment of recurrent, inoperable diffuse pediatric gliomas.

Proposed risk stratification model and treatment algorithm

Ryall et al. developed a comprehensive risk stratification model that incorporates patient age, tumor location, histopathologic features, and molecular profile. According to this model, pediatric gliomas are divided into three categories, namely low-risk, intermediate risk, and high risk, depending on the total score obtained after considering the above-mentioned clinical variables. Patients with a total score of (3-4 points) are treated conservatively, where they undergo gross total resection and subsequent observation (watchful waiting). Individuals with a score of 5-6 points are managed proactively through a combination of maximum safe or gross total resection, followed by conventional chemotherapy or targeted therapies. Careful close surveillance is done to detect any recurrence or aggressive behavior. Finally, high-risk patients with a total score of 7+ points are managed aggressively utilizing all possible therapeutic modalities, including maximum safe resection, multimodal conventional chemotherapeutic agents, and are subsequently enrolled in various clinical trials [73].

## Conclusions

In a nutshell, surgery remains the mainstay treatment for pediatric-type diffuse gliomas. However, not all tumors are located superficially, making their gross total resection tedious and, in certain instances, unachievable. In the past, various alternative therapeutic strategies had been employed to deal with recurrent and irresectable tumors, including conventional chemotherapy and radiotherapy, each of which has its own limitations and neurologic sequelae. With the advent of advanced ancillary techniques (immunohistochemistry, fluorescence in situ hybridization, droplet digital PCR, next generation sequencing, single-nucleotide polymorphism array, and nanostring ncounter and array methylation), precise molecular targets have been identified, opening a gateway to precision medicine and small molecule-based targeted therapy. There are, however, limitations in our knowledge regarding the adverse effects of new targeted therapies, raising serious concerns about their safety profiles and long-term patient outcomes. Numerous clinical trials are underway and are likely to provide a detailed account of the safety profile and feasibility of these yet enigmatic targeted therapies. Moving forward, a multidisciplinary approach combining molecular diagnostics, clinical acumen, and long-term follow-up will be essential to optimize treatment strategies and improve prognostic outcomes for pediatric patients with diffuse low-grade gliomas.

## References

[1] Ostrom QT, Price M, Ryan K (2022). CBTRUS statistical report: pediatric brain tumor foundation childhood and adolescent primary brain and other central nervous system tumors diagnosed in the United States in 2014-2018. Neuro Oncol.

[2] Aguilera D, Janss A, Mazewski C (2016). Successful retreatment of a child with a refractory brainstem ganglioglioma with vemurafenib. Pediatr Blood Cancer.

[3] Ostrom QT, de Blank PM, Kruchko C (2015). Alex's Lemonade Stand Foundation infant and childhood primary brain and central nervous system tumors diagnosed in the United States in 2007-2011. Neuro Oncol.

[4] Aisner DL, Newell KL, Pollack AG, Kleinschmidt-Demasters BK, Steinberg GK, Smyth LT, Vogel H (2014). Composite pleomorphic xanthoastrocytoma-epithelioid glioneuronal tumor with BRAF V600E mutation - report of three cases. Clin Neuropathol.

[5] Sturm D, Pfister SM, Jones DT (2017). Pediatric gliomas: current concepts on diagnosis, biology, and clinical management. J Clin Oncol.

[6] Louis DN, Ohgaki H, Wiestler OD (2007). The 2007 WHO classification of tumours of the central nervous system. Acta Neuropathol.

[7] Louis DN, Perry A, Burger P (2014). International Society Of Neuropathology--Haarlem consensus guidelines for nervous system tumor classification and grading. Brain Pathol.

[8] Ryall S, Tabori U, Hawkins C (2017). A comprehensive review of paediatric low-grade diffuse glioma: pathology, molecular genetics and treatment. Brain Tumor Pathol.

[9] Ferris SP, Goode B, Joseph NM (2016). IDH1 mutation can be present in diffuse astrocytomas and giant cell glioblastomas of young children under 10 years of age. Acta Neuropathol.

[10] Louis DN, Giannini C, Capper D (2018). cIMPACT-NOW update 2: diagnostic clarifications for diffuse midline glioma, H3 K27M-mutant and diffuse astrocytoma/anaplastic astrocytoma, IDH-mutant. Acta Neuropathol.

[11] Ellison DW, Hawkins C, Jones DT, Onar-Thomas A, Pfister SM, Reifenberger G, Louis DN (2019). cIMPACT-NOW update 4: diffuse gliomas characterized by MYB, MYBL1, or FGFR1 alterations or BRAF(V600E) mutation. Acta Neuropathol.

[12] Lehman NL (2016). Surgical neuropathology of focal epilepsies: textbook and atlas. J Neuropathol Exp Neurol.

[13] Wefers AK, Stichel D, Schrimpf D (2020). Isomorphic diffuse glioma has recurrent gene fusions of MYBL1 or MYB and can be distinguished from other MYB/MYBL1 altered gliomas based on a distinct morphology and DNA methylation profile. Neuro Oncol.

[14] Chiang J, Harreld JH, Tinkle CL (2019). A single-center study of the clinicopathologic correlates of gliomas with a MYB or MYBL1 alteration. Acta Neuropathol.

[15] Rigsby RK, Brahmbhatt P, Desai AB (2023). Newly recognized CNS tumors in the 2021 World Health Organization classification: imaging overview with histopathologic and genetic correlation. AJNR Am J Neuroradiol.

[16] Zhang R, Xu X, Zhou H, Yao D, Wei R, Muhammad S (2021). Pediatric angiocentric glioma with acute intracerebral hemorrhage: a case report with 36 months follow-up. Surg Neurol Int.

[17] Moreira DC, Qaddoumi I, Spiller S (2024). Comprehensive analysis of MYB/MYBL1-altered pediatric-type diffuse low-grade glioma. Neuro Oncol.

[18] Lellouch-Tubiana A, Boddaert N, Bourgeois M (2005). Angiocentric neuroepithelial tumor (ANET): a new epilepsy-related clinicopathological entity with distinctive MRI. Brain Pathol.

[19] Wang M, Tihan T, Rojiani AM (2005). Monomorphous angiocentric glioma: a distinctive epileptogenic neoplasm with features of infiltrating astrocytoma and ependymoma. J Neuropathol Exp Neurol.

[20] Marburger T, Prayson R (2011). Angiocentric glioma: a clinicopathologic review of 5 tumors with identification of associated cortical dysplasia. Arch Pathol Lab Med.

[21] Huse JT, Snuderl M, Jones DT (2017). Polymorphous low-grade neuroepithelial tumor of the young (PLNTY): an epileptogenic neoplasm with oligodendroglioma-like components, aberrant CD34 expression, and genetic alterations involving the MAP kinase pathway. Acta Neuropathol.

[22] Aguilar HN, Hung RW, Mehta V, Kotylak T (2012). Imaging characteristics of an unusual, high-grade angiocentric glioma: a case report and review of the literature. J Radiol Case Rep.

[23] Preusser M, Hoischen A, Novak K (2007). Angiocentric glioma: report of clinico-pathologic and genetic findings in 8 cases. Am J Surg Pathol.

[24] Mitani Y, Rao PH, Futreal PA (2011). Novel chromosomal rearrangements and break points at the t (6; 9) in salivary adenoid cystic carcinoma: association with MYB-NFIB chimeric fusion, MYB expression, and clinical outcome. Clin Cancer Res.

[25] Bergthold G, Bandopadhayay P, Ramkissoon L (2016). LG-02: MYB-QKI rearrangements in angiocentric glioma drive tumorigenicity through a tripartite mechanism. Neuro Oncol.

[26] Ozair A, Bhat V, Alisch RS (2023). DNA methylation and histone modification in low-grade gliomas: current understanding and potential clinical targets. Cancers.

[27] Almubarak AO, Alahmari A, Al Hindi H, AlShail E (2020). Angiocentric glioma of brainstem. Neurosciences (Riyadh).

[28] Georgescu M-M (2021). Multi-platform classification of IDH-wild-type glioblastoma based on ERK/MAPK pathway: diagnostic, prognostic and therapeutic implications. Cancers.

[29] Wang H, Tan C, Xu T, Li W (2021). Rapid progression of an IDH-wild type histological low-grade glioma harbouring TERT promoter mutation and diffuse CD34 expression: a case report. Folia Neuropathol.

[30] Yadav V, Zhang X, Liu J (2012). Reactivation of mitogen-activated protein kinase (MAPK) pathway by FGF receptor 3 (FGFR3)/Ras mediates resistance to vemurafenib in human B-RAF V600E mutant melanoma. J Biol Chem.

[31] Singh D, Joshi VP, Pattankar S (2024). Polymorphous low-grade neuroepithelial tumor of the young (PLNTY): scoping review of case reports and case series. Asian J Neurosurg.

[32] Chen W-X, Liu H-S, Yang S-D (2016). Reversible splenial lesion syndrome in children: retrospective study and summary of case series. Brain Dev.

[33] Johnson DR, Giannini C, Jenkins RB, Kim DK, Kaufmann TJ (2019). Plenty of calcification: imaging characterization of polymorphous low-grade neuroepithelial tumor of the young. Neuroradiology.

[34] Kurokawa M, Kurokawa R, Capizzano AA (2022). Neuroradiological features of the polymorphous low-grade neuroepithelial tumor of the young: five new cases with a systematic review of the literature. Neuroradiology.

[35] Bale TA, Rosenblum MK (2022). The 2021 WHO classification of tumors of the central nervous system: an update on pediatric low‐grade gliomas and glioneuronal tumors. Brain Pathol.

[36] Wang H, Zhu J, Zhu P, Luo C (2021). Angiocentric glioma: a case report and review of the literature. J Clin Neurosci.

[37] Fei X, Zhao J, Wei W (2022). Clinical, radiological, pathological features and seizure outcome with surgical management of polymorphous low-grade neuroepithelial tumor of the young associated with epilepsy. Front Oncol.

[38] Rao S, Goyal A, Johnson A, Sadashiva N, Kulanthaivelu K, Vazhayil V, Santosh V (2024). MAPK pathway alterations in polymorphous low-grade neuroepithelial tumor of the young: diagnostic considerations. Brain Tumor Pathol.

[39] Sabbagh MF, Janovitz T, Dias-Santagata D (2024). FGFR alterations in thyroid carcinoma: a novel class of primary drivers with significant therapeutic implications and secondary molecular events potentially mediating resistance in thyroid malignancy. Thyroid.

[40] Fabbri VP, Caporalini C, Asioli S, Buccoliero A (2022). Paediatric-type diffuse low-grade gliomas: a clinically and biologically distinct group of tumours with a favourable outcome. Pathologica.

[41] Van Maren E, Dankbaar J, Wesseling P (2024). T2-FLAIR mismatch: an imaging biomarker for children’s MYB/MYBL1-altered diffuse astrocytoma or angiocentric glioma. Am J Neuroradiol.

[42] Stone TJ, Merve A, Valerio F, Yasin SA, Jacques TS (2024). Paediatric low-grade glioma: the role of classical pathology in integrated diagnostic practice. Childs Nerv Sys.

[43] Thomas DL (2023). 2021 updates to the World Health Organization classification of adult-type and pediatric-type diffuse gliomas: a clinical practice review. Chin Clin Oncol.

[44] Louis DN, Perry A, Wesseling P (2021). The 2021 WHO classification of tumors of the central nervous system: a summary. Neuro Oncol.

[45] Sumdani H, Shahbuddin Z, Harper G, Hamilton L (2019). Case report of rarely described polymorphous low-grade neuroepithelial tumor of the young and comparison with oligodendroglioma. World Neurosurg.

[46] Ida C, Johnson D, Kollmeyer T (2019). Polymorphous low-grade neuroepithelial tumor of the young (PLNTY): genetic analysis confirms frequent MAPK pathway activation. Neuro Oncol.

[47] Lelotte J, Duprez T, Raftopoulos C, Michotte A (2020). Polymorphous low-grade neuroepithelial tumor of the young: case report of a newly described histopathological entity. Acta Neurol Belg.

[48] Gupta VR, Giller C, Kolhe R, Forseen SE, Sharma S (2019). Polymorphous low-grade neuroepithelial tumor of the young: a case report with genomic findings. World Neurosurg.

[49] Wisoff JH, Sanford RA, Heier LA (2011). Primary neurosurgery for pediatric low-grade gliomas: a prospective multi-institutional study from the Children's Oncology Group. Neurosurgery.

[50] Dodgshun AJ, Maixner WJ, Hansford JR, Sullivan MJ (2016). Low rates of recurrence and slow progression of pediatric pilocytic astrocytoma after gross-total resection: justification for reducing surveillance imaging. J Neurosurg Pediatr.

[51] Stokland T, Liu JF, Ironside JW (2010). A multivariate analysis of factors determining tumor progression in childhood low-grade glioma: a population-based cohort study (CCLG CNS9702). Neuro Oncol.

[52] Bandopadhayay P, Bergthold G, London WB (2014). Long-term outcome of 4,040 children diagnosed with pediatric low-grade gliomas: an analysis of the Surveillance Epidemiology and End Results (SEER) database. Pediatr Blood Cancer.

[53] Ater JL, Xia C, Mazewski CM (2016). Nonrandomized comparison of neurofibromatosis type 1 and non-neurofibromatosis type 1 children who received carboplatin and vincristine for progressive low-grade glioma: a report from the Children's Oncology Group. Cancer.

[54] Aquino VM, Fort DW, Kamen BA (1999). Carboplatin for the treatment of children with newly diagnosed optic chiasm gliomas: a phase II study. J Neurooncol.

[55] Dodgshun AJ, Maixner WJ, Heath JA, Sullivan MJ, Hansford JR (2016). Single agent carboplatin for pediatric low-grade glioma: A retrospective analysis shows equivalent efficacy to multiagent chemotherapy. Int J Cancer.

[56] Lassaletta A, Scheinemann K, Zelcer SM (2016). Phase II weekly vinblastine for chemotherapy-naïve children with progressive low-grade glioma: a Canadian pediatric brain tumor consortium study. J Clin Oncol.

[57] Merchant TE, Kun LE, Wu S, Xiong X, Sanford RA, Boop FA (2009). Phase II trial of conformal radiation therapy for pediatric low-grade glioma. J Clin Oncol.

[58] Packer RJ, Lange B, Ater J (1993). Carboplatin and vincristine for recurrent and newly diagnosed low-grade gliomas of childhood. J Clin Oncol.

[59] Saran FH, Baumert BG, Khoo VS, Adams EJ, Garré ML, Warrington AP, Brada M (2002). Stereotactically guided conformal radiotherapy for progressive low-grade gliomas of childhood. Int J Radiat Oncol Biol Phys.

[60] Indelicato DJ, Rotondo RL, Uezono H (2019). Outcomes following proton therapy for pediatric low-grade glioma. Int J Radiat Oncol Biol Phys.

[61] Merchant TE, Conklin HM, Wu S, Lustig RH, Xiong X (2009). Late effects of conformal radiation therapy for pediatric patients with low-grade glioma: prospective evaluation of cognitive, endocrine, and hearing deficits. J Clin Oncol.

[62] Grabenbauer GG, Schuchardt U, Buchfelder M (2000). Radiation therapy of optico-hypothalamic gliomas (OHG)-radiographic response, vision and late toxicity. Radiother Oncol.

[63] Erkal HŞ, Serin M, Çakmak A (1997). Management of optic pathway and chiasmatic-hypothalamic gliomas in children with radiation therapy. Radiother Oncol.

[64] Nageswara Rao AA, Packer RJ (2014). Advances in the management of low-grade gliomas. Curr Oncol Rep.

[65] Banerjee A, Jakacki RI, Onar-Thomas A (2017). A phase I trial of the MEK inhibitor selumetinib (AZD6244) in pediatric patients with recurrent or refractory low-grade glioma: a Pediatric Brain Tumor Consortium (PBTC) study. Neuro Oncol.

[66] Fangusaro J, Onar-Thomas A, Wu S (2020). A phase II re-treatment study of selumetinib for recurrent or progressive pediatric low-grade glioma (PLGG): a pediatric brain tumor Consortium (PBTC) study. Neuro Oncol.

[67] Cipri S, Del Baldo G, Fabozzi F, Boccuto L, Carai A, Mastronuzzi A (2023). Unlocking the power of precision medicine for pediatric low-grade gliomas: molecular characterization for targeted therapies with enhanced safety and efficacy. Front Oncol.

[68] Li T, Aihemaitiniyazi A, Zhang H (2025). Clinical characteristics and detection of MYB-QKI fusions in patients with angiocentric glioma. Neurol Sci.

[69] Chan E, Bollen AW, Sirohi D (2017). Angiocentric glioma with MYB-QKI fusion located in the brainstem, rather than cerebral cortex. Acta Neuropathol.

[70] Bandopadhayay P, Ramkissoon LA, Jain P (2016). MYB-QKI rearrangements in angiocentric glioma drive tumorigenicity through a tripartite mechanism. Nat Genet.

[71] Kumar D, Kiran F, Gener M, Kats A, Zhang L (2024). P118: Two cases of angiocentric glioma with MYB:: QKI fusion in a single institution. Genetics in Medicine Open.

[72] Nicolaides T, Nazemi KJ, Crawford J (2020). Phase I study of vemurafenib in children with recurrent or progressive BRAF(V600E) mutant brain tumors: Pacific Pediatric Neuro-Oncology Consortium study (PNOC-002). Oncotarget.

[73] Ryall S, Tabori U, Hawkins C (2020). Pediatric low-grade glioma in the era of molecular diagnostics. Acta Neuropathol Commun.

[74] Dai S, Zhou Z, Chen Z, Xu G, Chen Y (2019). Fibroblast growth factor receptors (FGFRs): structures and small molecule inhibitors. Cells.

